# Multi-meta-omics reveal distinct microbial genomic profiles and metabolic dysregulation in non-celiac gluten sensitivity

**DOI:** 10.1128/msphere.00856-25

**Published:** 2026-03-30

**Authors:** Kunal Dixit, Susheel Bhanu Busi, Anam Ahmed, Avinash Kshirsagar, Christian Jäger, Alka Singh, Varun Shah, Sunil D. Saroj, Vineet Ahuja, Paul Wilmes, Yogesh Shouche, Govind Makharia, Dhiraj Dhotre

**Affiliations:** 1 Symbiosis School of Biological Sciences, Symbiosis International (Deemed University) 29630, Pune, India; 2 Department of Food Science and Technology, University of California 8789, Davis, California, USA; 3 Luxembourg Center for Systems Biomedicine, University of Luxembourg 81872, Esch-sur-Alzette, Luxembourg; 4 UK Centre for Ecology and Hydrology 41865, Wallingford, Oxfordshire, United Kingdom; 5 Department of Gastroenterology and Human nutrition, All India Institute of Medical Sciences 28730 , New Delhi, India; 6 McMaster University 3710, Hamilton, Canada; 7 National Centre for Cell Science 29414, Pune, India; 8 Gujarat Biotechnology University 633303, Gandhinagar, India; 9 SKAN Research Trust 663357 , Bengaluru, India; Kansas State University, Manhattan, Kansas, USA

**Keywords:** non-celiac gluten sensitivity, metagenomics, metabolomics, gut microbiome, irritable bowel syndrome, gluten-free diet

## Abstract

Non-celiac gluten sensitivity (NCGS) is an emerging diagnosis, and its symptoms overlap with irritable bowel syndrome (IBS). The gut microbiome is likely to play a role in the pathogenesis of NCGS. We analyzed the gut microbiome in patients with NCGS and in patients with IBS, using shotgun metagenomics and metabolomics of fecal samples. Analyses of taxonomic and functional microbial diversity revealed a higher abundance of methanogenic archaea, such as *Methanobrevibacter filiformis*, *Methanobrevibacter boviskoreani*, *Methanosphaera stadtmanae*, and a higher fold change in urea, uridine 5-monophosphate, and adenosine monophosphate in patients with NCGS compared to patients with IBS, who showed higher fold changes in metabolites gamma-aminobutyric acid and lactic acid. Furthermore, pangenome and metabolome analyses revealed disease-specific gene clusters, as well as genomic and metabolic features differentiating NCGS from IBS. While patients with NCGS did not show lower potential for gluten degradation, a lower synthetic potential for fructan beta-fructosidase was found in them. The present study provides an extensive analysis of taxonomic, genomic, and metabolic features that may play a role in the pathogenesis and symptom development in patients with NCGS.

**IMPORTANCE:**

Non-celiac gluten sensitivity (NCGS) is an emerging diagnosis with symptoms that overlap with irritable bowel syndrome (IBS). Using shotgun metagenomics and metabolomics, we report deeper insights into the microbiome profile, including viral and archaeal diversity, lower fructan degradation potential, the differential abundance of metabolites, and genomic features of gut bacteria in patients with NCGS. Understanding the microbiome associated with this disorder may shed light on the possible role of the microbiome in the pathophysiology of NCGS.

## INTRODUCTION

Non-celiac gluten sensitivity (NCGS) is an emerging disorder caused due to ingestion of wheat or its protein called gluten (1, 2). NCGS is manifested by both gastrointestinal and extraintestinal symptoms, such as abdominal pain, altered stool frequency, nausea, headache, brain fog, tingling and numbness, fatigue, and musculoskeletal pain (3, 4). A systematic review has reported a global prevalence of 10% with marked regional variation, being more common in developed countries (5). The wide range of prevalence reported for this gluten spectrum disorder is due to the different definitions applied for NCGS categorization across studies. In contrast to celiac disease, there is no diagnostic biomarker, and the diagnosis of NCGS is currently established by the presence of clinical response to a gluten-free diet (GFD) and recurrence of symptoms on the resumption of gluten in the diet (6). Furthermore, the clinical features of NCGS overlap with those of patients with irritable bowel syndrome (IBS), and many such patients are misdiagnosed as IBS patients.

While symptoms of NCGS are dependent on ingestion of wheat, it is proposed that the disease may occur either because of high fermentable oligo-, di-, monosaccharides, and polyols (FODMAP) contents of wheat or due to gluten in it. The proposed mechanism for occurance of symptoms in patients with NCGS is an innate immune response to gliadin peptides, hypersensitivity of the intestine, and intestinal motility disorder. A number of reports suggest gut dysbiosis in patients with IBS (7–10). It is plausible that gut microbiota handle gluten in such a way as to create peptides that induce innate immune response and intestinal dysmotility in patients with NCGS.

There is limited knowledge about the gut microbiota and metabolites in patients with NCGS; thus, it is imperative to study this gluten spectrum disorder with a multi-omic approach to delineate the differences and also to understand the commensal or pathogenic role of the microbiota in them.

To address the current gaps in knowledge, we recruited patients with IBS and NCGS based on standard criteria to map differences in the microbiome structure, functions, and metabolome profiles between the two disorders. We also estimated the effect of GFD on patients with NCGS by comparing NCGS patient samples pre- and post-GFD. We further examined the load of microbially coded genes responsible for gluten degradation between these disorders, as lower gluten degradation has already been found in other gluten spectrum disorders. We identified differences in microbiome-based taxonomic as well as functional profiles for NCGS and IBS patients.

## RESULTS

### Recruitment of patients and study overview

**Fig 1:**
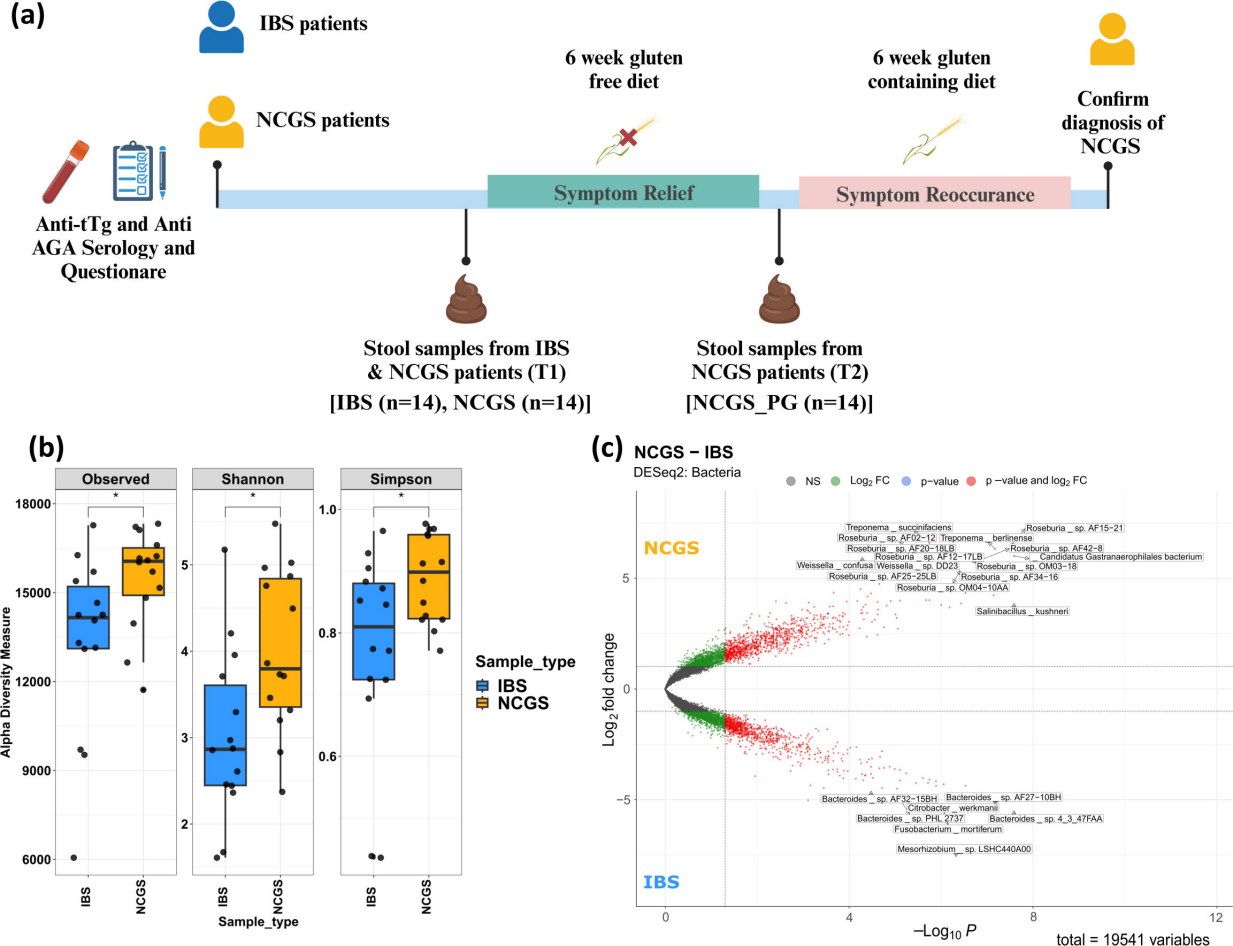
Bacterial diversity varies between NCGS and IBS patients. (**a**) Study design scheme depicting patient recruitment and sample collection. (**b**) Box plot illustrating the bacterial alpha diversity measures of NCGS and IBS patient stool samples. (**c**) Volcano plot presenting the differentially abundant bacterial species in NCGS and IBS patients. Comparisons shown are between IBS and pre-GFD NCGS only.

The details of patient recruitment criteria, symptom score, and details for patient response to GFD intervention have been published elsewhere (11). To map the taxonomic diversity, functional potential, and metabolite profiles of the microbiome, we performed shotgun metagenomics and metabolomics on a total of 42 stool samples collected from patients with IBS (*n* = 14), patients with NCGS before GFD (*n* = 14; NCGS), and after GFD (*n* = 14; NCGS patients post-GFD [NCGS_PG]; Fig. 1a). Whole-genome metagenome sequencing yielded a total of 7.0 × 10^7^ (±1.0 × 10^7^ SD) reads per sample. Adapter trimming discarded 2.7% of the total reads, and on average, 19.10% of the reads were found to be of human origin and therefore discarded. The sequencing characteristics, pre- and post-quality control, and after assembly are highlighted in Data S1. Additionally, for the IBS, NCGS, and NCGS_PG groups, a total of 406, 515, and 526 metagenomically assembled genomes (MAGs) were obtained after assembly and binning, out of which 170, 227, and 225 MAGs, respectively, had completeness >70% and contamination <10%. Quality data for MAGs are provided in the Data S2. A total of 171 metabolite features were profiled with gas chromatography-mass spectrometry (GC-MS), of which 82 were identified. Additionally, we could profile and identify a total of 87 metabolites using liquid chromatography-mass spectrometry (LC-MS) in stool samples of IBS patients and NCGS patients. The list of all metabolites detected in the stool samples by GC-MS and LC-MS is provided as Data S3.

### Metagenomics reveals diversity differences across groups

**Fig 2:**
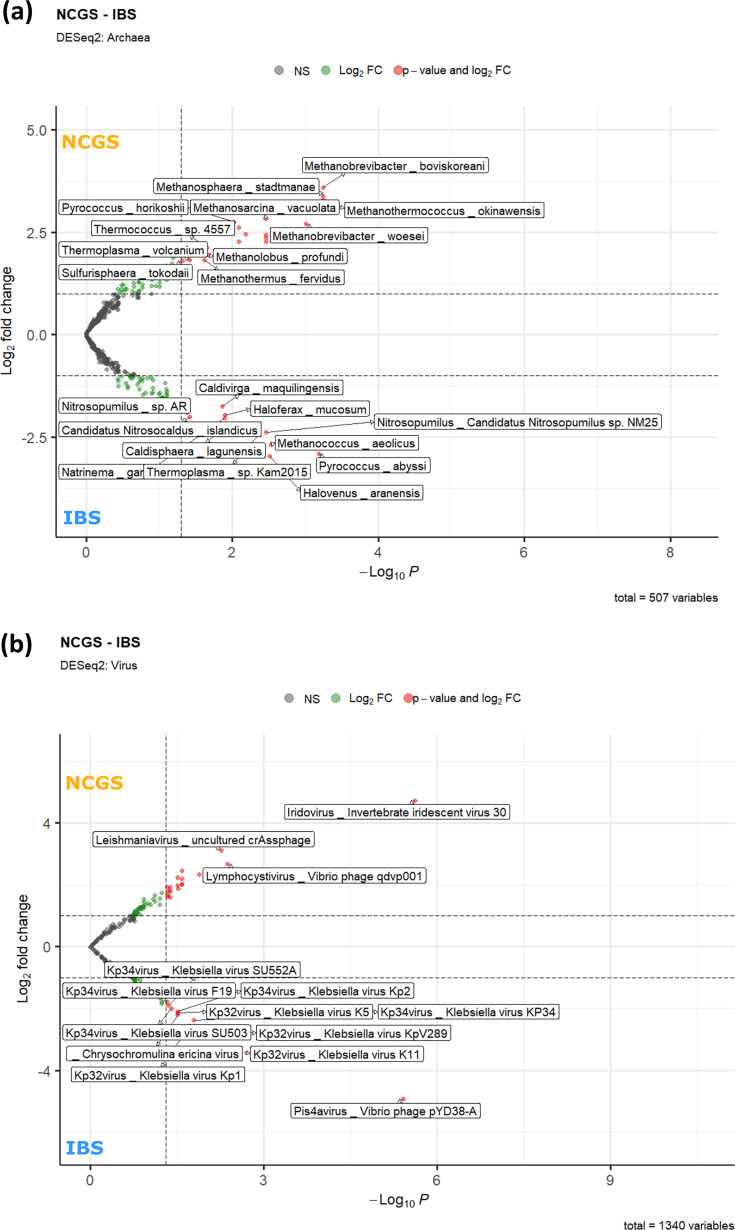
NCGS and IBS have differential archaeal and viral community structure. (**a**) Volcano plot depicting differentially abundant archaeal diversity between study groups based on DeSeq2 analysis. (**b**) Volcano plot depicting differentially abundant viral diversity between study groups based on DeSeq2 analysis. Comparisons shown are between IBS and pre-GFD NCGS only.

Microbial diversity was captured based on the metagenomic read mapping against the comprehensive Kraken database (see Materials and Methods). Comparative microbiome analyses were performed for IBS patients and NCGS patients (before GFD) as well as NCGS samples before and after GFD (NCGS_PG). The diversity spanning three broad taxonomic clades (bacteria, archaea, and viruses) was assessed using alpha diversity indices (including richness, Shannon, and Simpson), alongside differential taxa analysis. All three alpha diversity metrics showed higher bacterial diversity in the NCGS group compared with the IBS group. Alpha diversity measures suggested higher bacterial diversity in the NCGS group compared to IBS patients (Fig. 1b). DESeq2 analyses (Wald test with Benjamini-Hochberg correction) revealed significant differences in taxa from all three taxonomic groups. The genera *Roseburia*, *Treponema*, *Weissella*, alongside *Salinibacillus kushneri*, were found in greater numbers in patients with NCGS. On the other hand, genus *Bacteroides*, along with *Fusobacterium mortiferum* and *Citrobacter werkmanii*, were higher in IBS patients (Fig. 1c; Data S2). Moreover, the differentially higher profile of NCGS archaea was mainly characterized by methane-producing species *Methanosphaera*, *Methanobrevibacter*, *Methanosarcina*, *Methanothermococcus*, *Methanothermus*, etc. (Fig. 2a). Subsequently, we assessed the virome profiles of NCGS and IBS patients and found that they largely overlapped (Fig. S1). However, differentially higher amounts of *Iridovirus*, *Leishmaniavirus*, and *Lymphocystivirus* were found in NCGS patients; whereas, multiple phages targeting *Klebsiella* were found differentially abundant in IBS patients (Fig. 2b). The complete list of differentially abundant taxa can be found as part of Data S2.

### Functional diversity differences between NCGS and IBS patients

**Fig 3:**
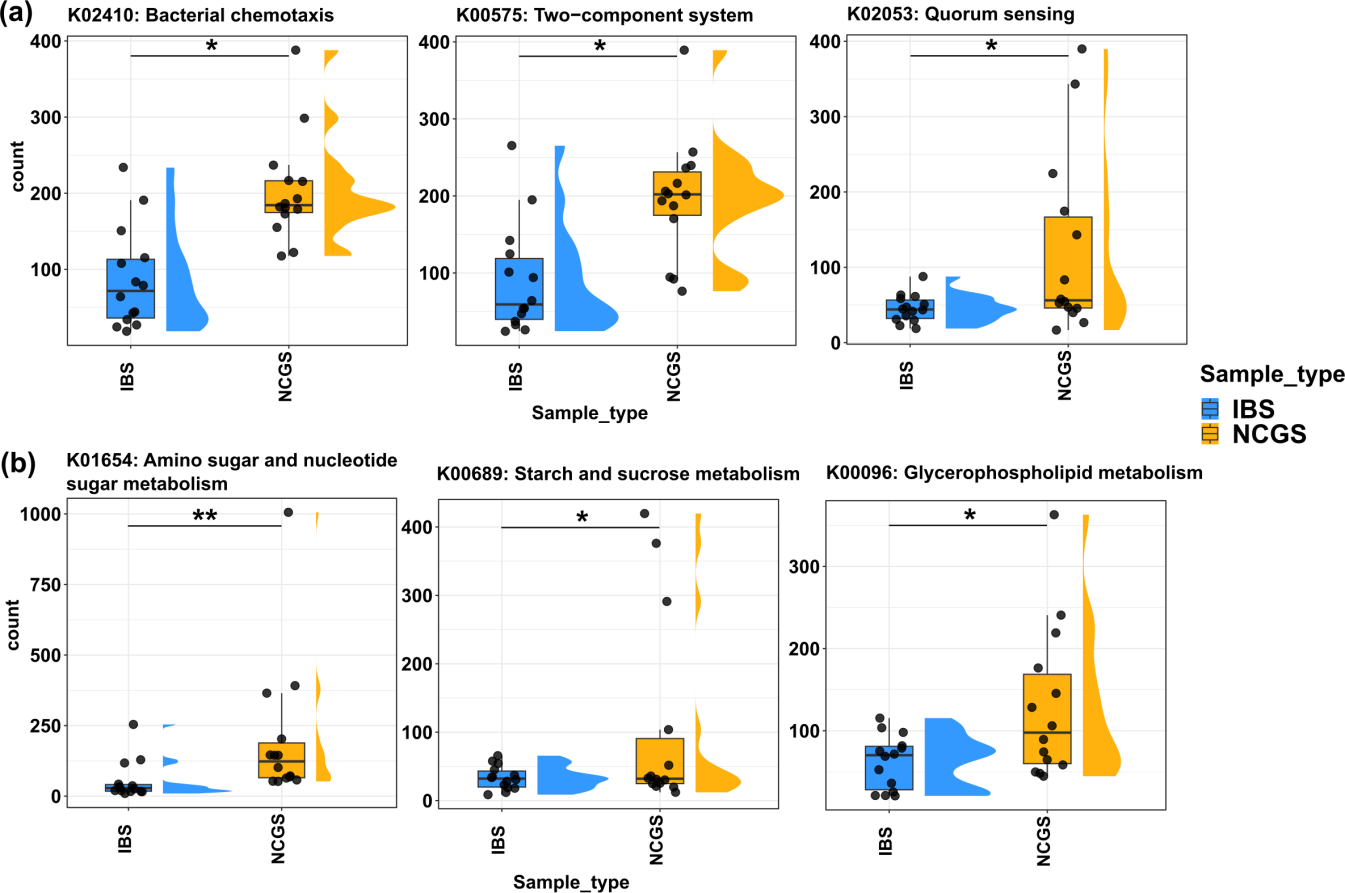
Microbial functional potential of NCGS and IBS patients is significantly different. KOs responsible for (**a**) bacterial communication and (**b**) sugar metabolism were found significantly higher in patients with NCGS (*P*adj < 0.05). Comparisons shown are between IBS and pre-GFD NCGS only.

To complement the taxonomic analyses, we assessed the functional potential of the metagenomes, i.e., of the assembled contigs, by mapping them to the Kyoto encyclopedia of genes and genomes (KEGG), carbohydrate-active enzyme (CAZyme), and Pfam databases, respectively. A total of 14,014 KEGG Orthologs (KOs), 338 CAZymes, and 8,988 protein families were identified in total, of which 305 KOs, 16 CAZymes, and 178 protein families were differential between NCGS and IBS (DESeq2 analysis; Wald test; Fig. S2; Data S4). A higher number of KO and protein families was observed in NCGS patients compared to IBS patients; however, the differences were not statistically significant. Interestingly, NCGS patients possessed a significantly higher abundance of K02410, K00575, and K02053 (*P*adj. 0.015, 0.030, and 0.036, respectively), associated with microbial communication, such as bacterial chemotaxis, the two-component system, and quorum sensing. Moreover, significantly higher levels of carbohydrate-active enzymes in NCGS patients were reflected by higher levels of K01654, K00689, and K00096 (*P*adj. 0.004, 0.015, and 0.040, respectively), which are responsible for sugar metabolism (Fig. 3a and b).

### Effect of gluten-free diet on patients with NCGS

NCGS patients were kept on GFD for 6 weeks to map microbiome changes as a response to the intervention. The results for NCGS samples before and after GFD are mainly presented as part of the supplemental data. Kraken-based taxonomic annotation identified a total of 252 bacterial species and 19 viruses that were differentially abundant between NCGS and NCGS_PG groups. No archaeal species were found to be significantly different between these groups. The complete list of differentially abundant taxa is present as Data S2. In case of differences in the functional features in NCGS patients, no significant differences were reported in the overall diversity measures (observed features and Fisher index; *P* > 0.05) as an effect of GFD (Fig. S3a to c); however, 54 KOs, 16 CAZymes, and 26 protein families were found significantly different between NCGS and NCGS_PG groups (Fig. S3d to f and Data S4).

### Gluten degradation potential in NCGS as compared to IBS patients

**Fig 4:**
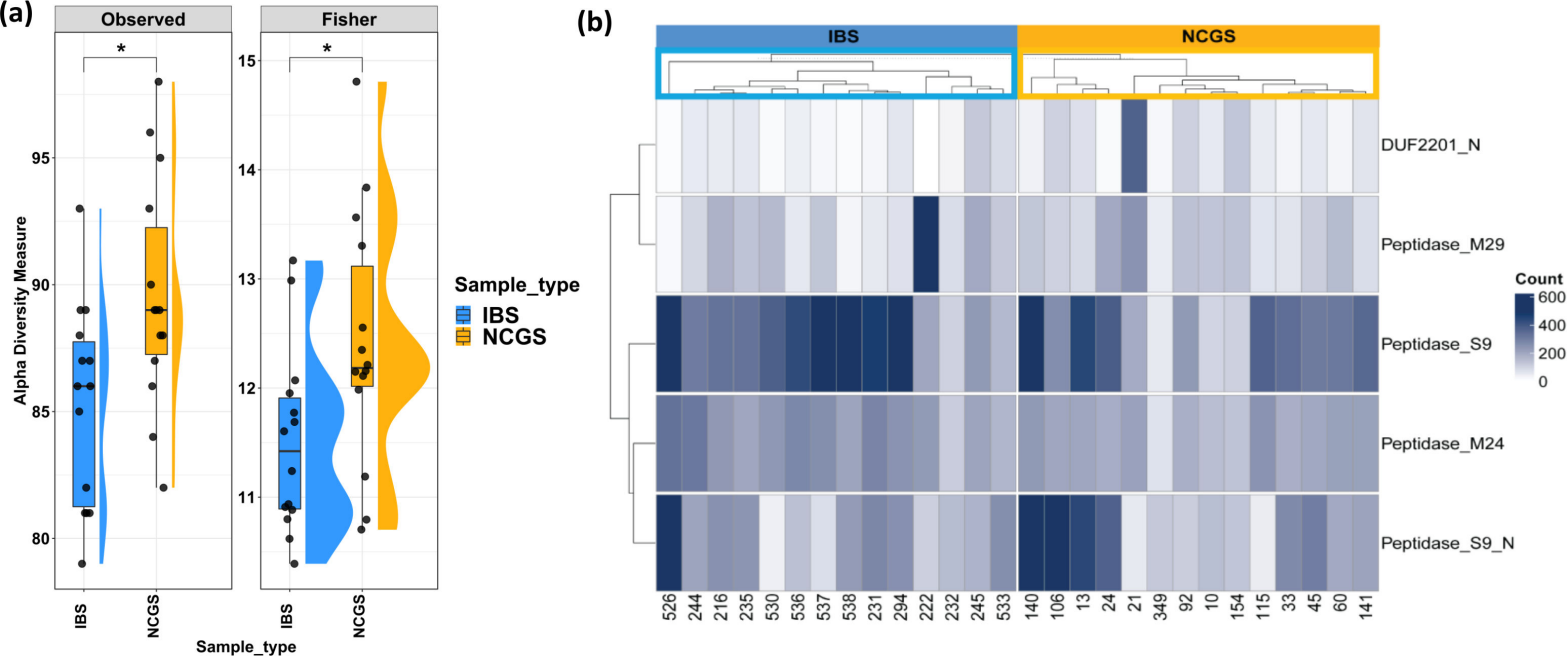
Protease profiles of NCGS and IBS patients. (**a**) Plot presenting higher alpha diversity of proteases in patients with NCGS. (**b**) Heatmap showing similar gluten degradation potential in both NCGS and IBS patients. Comparisons shown are between IBS and pre-GFD NCGS only.

Gluten-degrading proteolytic enzymes are speculated to correlate with symptom development in gluten spectrum disorders (12, 13). Interestingly, NCGS microbiomes encoded significantly higher proteases compared to IBS (Fig. 4a; *P*adj. < 0.05, Wilcoxon’s ranked sum test). Enzymes capable of degrading gluten were screened for their total numbers between the two perturbations and further mapped in the various pangenomes to test the involvement of differentially abundant taxa in gluten degradation. Microbial gluten degradation potential was assessed based on levels of previously reported gluten-degrading enzymes. These included putative metallopeptidase domain (DUF2201_N), thermophilic metalloprotease (peptidase_M29), prolyl oligopeptidase (peptidase_S9), prolyl oligopeptidase, N-terminal beta-propeller domain (peptidase_S9_N), and metallopeptidase family M24 (peptidase_M24). NCGS and IBS patients were found to have similar microbial gluten degradation potential based on the type and distribution of the enzymes. Importantly, a hierarchical clustering approach revealed distinct profiles separating the NCGS and IBS groups based on the abundance of these gluten-degrading enzymes (Fig. 4b).

### Pangenome analysis revealed NCGS-specific gene clusters

**Fig 5:**
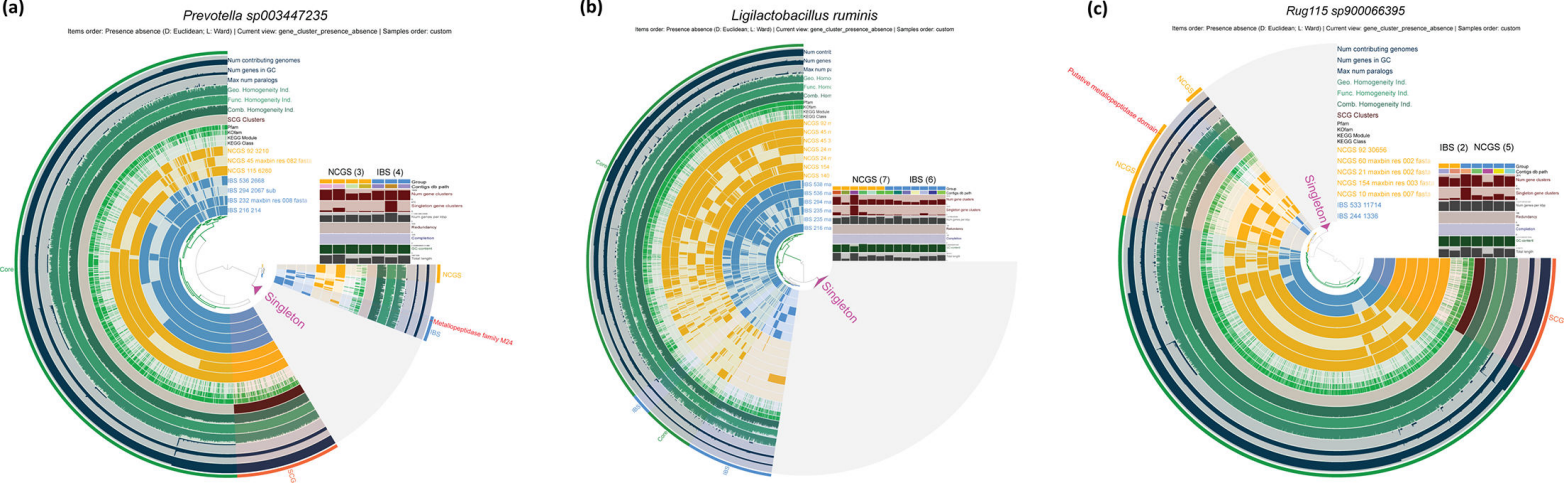
Pangenome analysis of metagenomically assembled genomes. (**a**) Pangenome of *Prevotella sp003447235* shows IBS and NCGS-specific gene clusters. (**b**) Pangenome of *Ligilactobacillus ruminis* shows IBS-specific gene clusters. (**c**) Pangenome of *RUG115 sp900066395* shows NCGS-specific gene clusters. Comparisons shown are between IBS and pre-GFD NCGS only.

To further understand the taxa contributing to the observed functional differences between the NCGS and IBS groups, we assessed the differential abundance of the medium-to-high-quality MAGs generated from the metagenomes (14). These analyses revealed 40 differentially abundant MAGs between NCGS and IBS. These included but were not limited to *Prevotella sp003447235*, *Ligilactobacillus ruminis*, *RUG115 sp900066395* (Data S5), which were further used for pangenome analyses to map the species-specific gluten degradation potential and explore differences in the group-specific gene clusters (Fig. 5a to c). In addition to earlier reported protein families with gluten degradation potential, we investigated disorder-specific gene clusters responsible for gluten degradation using pangenome analysis for these three species. We found that these three species had the potential for gluten degradation in both the NCGS and IBS groups, although no significant differences in their overall abundances were observed. Pangenome analysis revealed gene clusters on the genome of *Ligilactobacillus ruminis* that were unique to IBS. In contrast, the genomes of *RUG115 sp900066395* from NCGS patients had gene clusters unique to this disorder and encoded two additional putative metallopeptidase domains. Meanwhile, *Prevotella sp003447235* was found to possess unique gene clusters for both NCGS and IBS groups, with two metallopeptidase family “M24” gene cluster hits mapped specifically to IBS. A list of group-specific gene clusters in differential species pangenomes is provided in Data S5.

### Fructan degradation in NCGS

**Fig 6:**
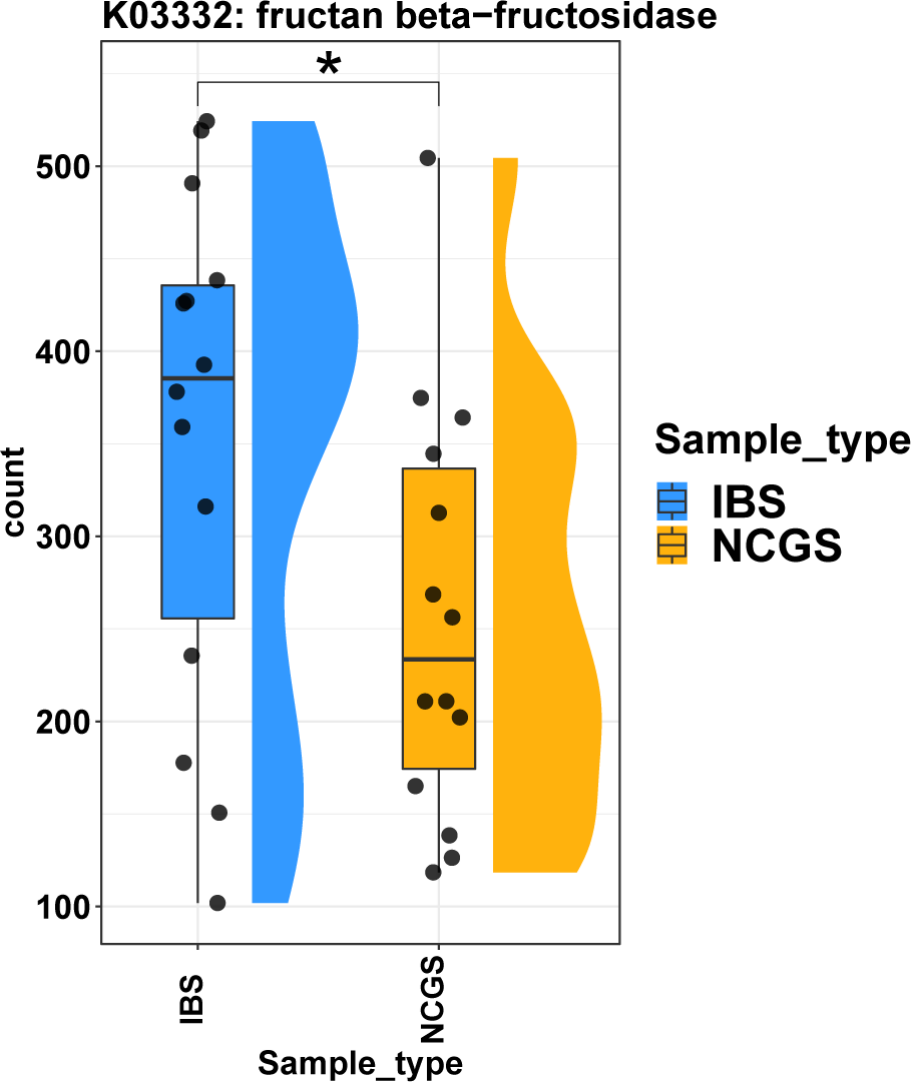
Lower abundance of fructan beta-fructosidase in patients with non-celiac gluten sensitivity. Comparisons shown are between IBS and pre-GFD NCGS only.

In addition to the gluten degradation capacity, microbial fructan degradation was tested in NCGS patients because fructan is increasingly suspected of being a probable cause of symptoms in NCGS patients (15). To understand if the potential for fructan degradation varied between NCGS and IBS, we extracted the relevant gene abundance information from the individual metagenomes across all samples. We found that the genes mapping to the KO K03332, encoding fructan beta-fructosidase involved in fructan hydrolysis, were observed at significantly lower levels in patients with NCGS (Fig. 6; *P*adj < 0.05, Wilcoxon rank-sum test) than in IBS. The median transcripts per million (TPM) abundance and IQR for the NCGS and IBS groups for this gene were 233.58 (174.37–336.66) and 385.43 (255.69–435.59), respectively.

### Metabolome profiles in patients

**Fig 7:**
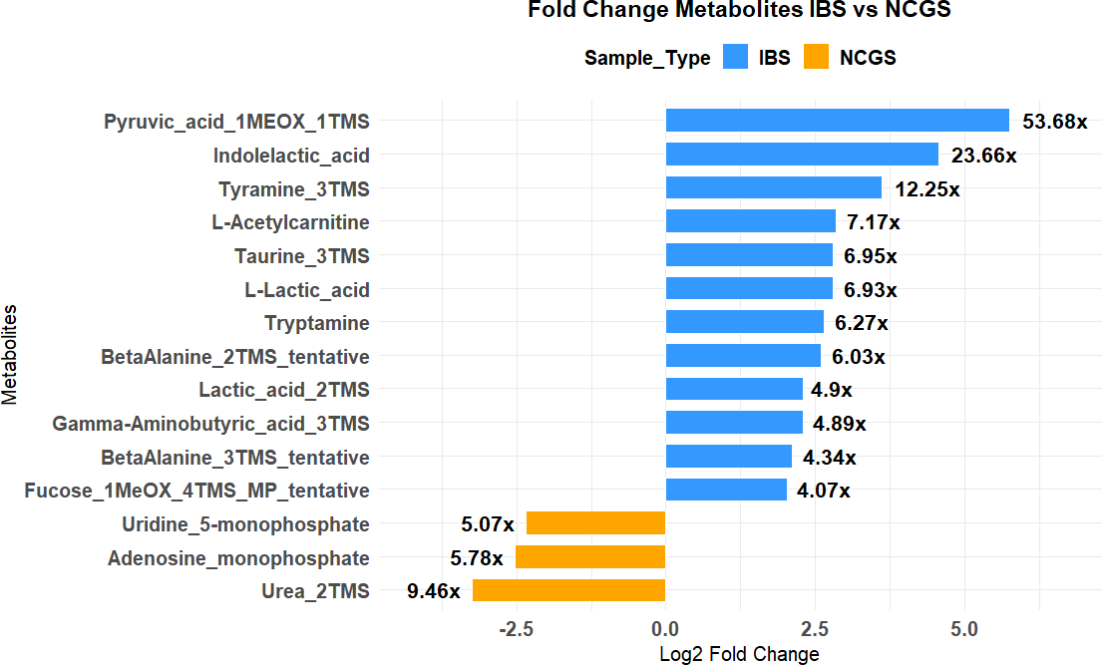
Fold change metabolite profile in NCGS and IBS patients. Bar plot showing fold changes for metabolites (log2 fold change > 2 are shown) identified using GC-MS and LC-MS analysis. The *y*-axis presents metabolites, the *x*-axis presents log2 fold change, and the actual fold changes are indicated on the respective bars. Comparisons shown are between IBS and pre-GFD NCGS only.

Glucose, carbonic acid, and glutamic acid were the most highly abundant metabolites found with GC-MS analysis in both NCGS and IBS patient stool samples and were similar between the NCGS and IBS patients. Diethylglycol, 5-aminovaleric acid, and lactic acid were found to be more abundant in IBS patients compared to NCGS patients. The principal coordinate analysis (PCoA) revealed that the stool metabolite profiles between NCGS and IBS patients, as well as NCGS patients before (NCGS) and after GFD (NCGS_PG), were in fact overlapping (Fig. S4). MetaboAnalyst 6.0 was used to find the fold changes in the metabolite profiles between NCGS and IBS patients. A total of 15 metabolites were found with log2 fold change greater than 2, which include uridine 5-monophosphate, adenosine monophosphate, and urea at higher fold changes in NCGS patients and metabolites such as gamma-aminobutyric acid, lactic acid, indolelactic acid, fucose, etc. at higher fold changes in IBS patients, although the differences were not statistically significant (Fig. 7).

## DISCUSSION

The prevalence of NCGS worldwide and the increasing evidence of its associations with the gut microbiome call for a comprehensive exploration of the microbiome associated with this gluten spectrum disorder (1, 2). Additionally, the highly overlapping manifestations and the lack of biomarkers call for a multi-omic analysis of the gut microbiome (16). Several reports suggest that the gut microbiome has a critical influence on NCGS, in addition to nutritional and environmental confounders (17–19). The present study provides clear findings, as the sampling strategy was aimed at excluding false-positive NCGS patients. We used shotgun metagenomics and metabolomics to compare the microbiome of 42 stool samples from IBS, NCGS, and NCGS post-GFD patients, revealing differences between taxonomic profiles, genomic features, and metabolites. The current study is the first extensive report in the context of NCGS that includes differential diversity across archaea and viruses besides bacteria as well as genomic potential and metabolome of stool samples. A recent report by Mohammadzadeh et al. (20) has highlighted that archaea, a key component of the human gut microbiome, remain understudied in terms of their pathogenicity (20). In our study, several methanogenic archaea, such as *Methanobrevibacter filiformis*, *Methanosarcina vacuolata*, *Methanobrevibacter boviskoreani*, and *Methanosphaera stadtmanae*, were found significantly more frequently in NCGS patients. *Methanobrevibacter* species are the main representatives of methanogens in the human system and are known to modulate host nutrient degradation. Several studies have demonstrated the presence of *Methanobrevibacter smithii* in patients with IBS, infectious endocarditis, and Parkinson’s disease, and it has been associated with high methane emission (20). Interestingly, we found differentially higher abundances of *Methanobrevibacter filiformis*, *Methanobrevibacter boviskoreani,* and *Methanobrevibacter woesei* in NCGS patients. *Methanosphaera stadtmanae* has also been previously detected in humans and is known to utilize acetate for methane production. It has been reported that higher methane synthesis affects intestinal transit time in IBS patients (21). It is plausible that a similar phenomenon may be responsible for the constipation in NCGS patients.

Furthermore, proteases are known to influence intestinal barrier functions, alter bowel habits, change visceral sensitivity, and immune response (22). In this context, the higher total protease levels in NCGS patients are also of interest. Higher concentrations of proteases in stool and biopsy specimens have previously been reported in patients with IBS and have been suggested to play a role in pathophysiology (22). There are no reports investigating proteases in NCGS patients. Here, we found significantly higher levels of proteases in NCGS patients compared to IBS patients, suggesting that they may affect the intestinal barrier and bowel habits of these patients, leading to the complex symptomatology observed in NCGS. Increased proteolysis could be due to the release of cysteine, serine, and metalloproteases by intestinal bacteria (22). Interestingly, some of these enzymes are known for their gluten-degrading activity and are suspected to have therapeutic potential in celiac disease, as lower levels of these enzymes have been reported in CeD patients (12, 23). We observed no significant difference in the levels of previously reported gluten-degrading enzymes between the study groups, suggesting that both disorders have similar gluten-degrading potential. This is further evidence that symptoms in NCGS patients may be due to a dietary factor other than gluten. A study by Skodje et al. (15) recently reported that fructans, a type of FODMAP, trigger symptoms in self-reported NCGS patients rather than gluten (15). Moreover, apart from GFD, a low-FODMAP diet has been shown to improve physiology in terms of reducing intraepithelial lymphocytes and increasing mucin-producing cells in NCGS patients (24). In parallel, we have observed in patients with NCGS a significantly lower abundance of fructan beta-fructosidase (K03332), which is primarily responsible for the hydrolysis of terminal beta-D-fructofuranose residues in fructans.

Pangenome analysis revealed NCGS- and IBS-specific gene clusters on the genomes of *Prevotella sp003447235* and *Rug115 sp900066395*. The appearance of additional genes encoding the gluten-degrading metallopeptidase S4 family in the IBS-specific cluster and of the putative metallopeptidase domain in the NCGS-specific clusters of these organisms may indicate their specific adaptability to the NCGS and IBS patient groups. In addition, pangenome analyses revealed genes encoding glycosyltransferase family 2, group 1, and WbsX, along with rhamnosyltransferase (EC: 2.4.1), which are present in the NCGS-specific gene cluster of *Rug115 sp900066395*. These proteins were previously found in the genome of *Ruminococcus gnavus* and play a role in the production of complex glucorhamnan polysaccharide, an inflammatory polysaccharide that induces TNFα, an inflammatory cytokine in patients with Crohn’s disease (25). Further validation is essential for the presence of this inflammatory molecule in NCGS patients and its link to the symptoms associated with the disorder.

Metabolome profiles of gluten spectrum disorders are limitedly investigated in previous studies to obtain biomarkers. Jacobs et al. (9) have reported that IBS can be characterized by lower gentisate and hydrocinnamate as well as higher tyramine, which was also found to be true in the present study. Also, they found that diarrhea-predominant IBS differs from constipation-predominant IBS in terms of increased bile acids, polyamines, malate, and fumarate (9). While stool metabolome profiles of NCGS and IBS patients appear similar for the most part, we identified a few metabolite targets with high fold change between the disorders. Urea has been found to cause intestinal barrier dysfunction when present in higher than clinically relevant concentrations. Higher amounts of urea have been reported to cause dissipation of transepithelial electrical resistance, causing loss of tight junction proteins (26). On the other hand, a higher concentration of pentoses in NCGS patients could be indicative of impaired metabolism.

We acknowledge that a caveat of our findings is the lack of a control group of healthy individuals without any NCGS or IBS manifestation. Interestingly, an earlier report by Mazcorro et al. (27) highlighted the taxonomic differences between healthy controls and NCGS patients (27). They reported significantly higher amounts of *Ruminococcaceae* in NCGS patients along with *Saccharibacteria, Actinobacillus*, and *Finegoldia*. In contrast, lower *Sphingobacterium* counts were found in healthy controls (27). Another study comparing NCGS with the microbiota of healthy individuals reported higher *Peptostreptococcaceae* and lower *Porphyromonadaceae* abundances in patients with NCGS (28). Though these are important findings, as highlighted previously, the diagnosis of NCGS is not straightforward, necessitating the need to develop key taxonomic and functional biomarkers for classifying and differentiating the two disorders. Another limitation of the current study is not taking IBS subtype classification into account for subtle variations within the microbiome (9). However, the current study focused on the overall differences between NCGS and IBS, given the possible misclassification of the two disorders, and a study with a larger sample size is required to track if IBS subtypes vary in microbiome composition as compared to NCGS patients. Our collective findings, including taxonomic, functional capacity, and the metabolomic information, therefore, provide a platform for leveraging key multi-omic modalities. These will not only allow for the classification and identification of NCGS but also will enable the understanding of underlying microbially mediated mechanisms in disease etiology.

## MATERIALS AND METHODS

### Patient recruitment

Patients were recruited from the outpatient clinic of the Department of Gastroenterology and Human Nutrition at the All India Institute of Medical Sciences (New Delhi). Patients were screened for IBS using the Rome IV criteria and the Bristol stool chart. Individuals with regular alcohol consumption (>20 g/week), smokers (>5 cigarettes or bidis/week), patients who had undergone abdominal surgery, intestinal resection, active nonsteroidal anti-inflammatory drugs usage, antibiotics, or prebiotics within 8 weeks, and patients with malabsorption syndromes (tropical sprue, giardiasis, or COVID) were excluded from the study. Routine screening tests were performed in all patients with IBS, such as microscopic examination of the stool (to rule out parasitic infections), fecal occult blood test, and routine hematologic and biochemical tests. Patients were also tested for AGA IgA and IgG titers >30 U/mL (QUANTA Lite Gliadin IgA, IgG). Celiac disease was ruled out in gluten-sensitive patients with negative anti-tTG IgA (QUANTA Lite R h-tTG IgA) and villous atrophy using histology of small intestinal biopsy. Gluten-sensitive patients were treated with a GFD intervention for 6 weeks. Patients who responded to GFD (>30% decrease in symptoms) were maintained on their regular (gluten containing) diet to confirm NCGS diagnosis if symptoms recurred. Data for response to GFD and symptom scores have been published elsewhere (11). Fecal samples were collected from IBS and gluten-sensitive patients. At a second time point, stool samples were collected from gluten-sensitive patients who responded to GFD after a 6-week intervention (Fig. 1a). All samples were frozen and stored at −80°C until further processing. More details for the patient recruitment and symptom data are published elsewhere (11).

### DNA extraction and metagenomic sequencing

Forty-two stool samples were collected from IBS patients and NCGS patients (before and after GFD). The QIAamp 96 PowerFecal QIAcube HT kit (QIAGEN, Valencia, CA, USA) was used for fecal DNA extraction using the QIAcube HT liquid handler system. Spectrophotometric methods NanoDrop ND-1000 spectrophotometer (NanoDrop Biotechnologies, USA) and Qubit fluorometer (Thermo Fisher Scientific, USA) were used to quantify the extracted DNA. DNA integrity was checked using agarose gel electrophoresis. The extracted DNA was diluted to 100 ng/µL and stored at −20°C until further use.

Sequencing was outsourced to Medgenome Labs, India. The DNA samples were subjected to random shotgun sequencing. Metagenome library preparation was performed using the KAPA DNA HyperPrep kit with nine PCR cycles. Ultrasonication was used to shear the DNA, followed by a sequence of enzymatic treatments. The next step was ligation of indexed adapters followed by purification with reversible immobilization using SPRI beads (Beckman Coulter Lifesciences, USA). Prepared libraries were sequenced on the Illumina HiSeqX platform to generate 10 gigabytes of data per sample using paired-end shotgun sequencing with 150 × 2 chemistry. Raw sequencing output was delivered in FASTQ format, with standard Illumina run-quality metrics indicating ≥75% of bases at Q30 or higher.

### Metagenomic data processing

Pre-processing of the above metagenomic data, genome assembly, as well as taxonomic and functional annotations with various databases, were done using the Integrated Meta-omics Pipeline (IMP) version 3 (29). Pre-processing included adapter trimming and subsequent filtering of *Homo sapiens* DNA (hg38) to obtain only microbiome-based gene sequences and functions. The next step was assembly using MEGAHIT (version 2.0) (30). A consensus approach including MaxBin (31) and MetaBat (32) was used to reconstruct MAGs, followed by selection of a non-redundant collection of MAGs using DASTool with a score threshold of 0.7 (33). CheckM v1.16 was used to assess the quality of MAGs, and taxonomy was assigned using the GTDB toolkit (34). IMP-based annotation of proteins (Pfam) (35), metabolic pathways (KEGG and MetaCyc) (36, 37), and carbohydrate-active enzymes (dbCAN) (38) was performed using contigs. The gene counts for these features were obtained by mapping the pre-processed reads (trimmed, quality-filtered, and human-reads removed) to the contigs and subsequently using featureCounts to obtain gene-specific abundances (39). To address the differences in sequencing depth per sample and variability in contig characteristics, we employed the TPM method for normalization, which accounts for the sequencing depth and the length of each identified gene. Read-based microbial taxonomic identification with Kraken (v2.1.0) (40) was also performed for metagenome data. For the read-based taxonomy, the maxikraken database available at https://lomanlab.github.io/mockcommunity/mc_databases.html was used. Specifically, the maxikraken2_1903_140GB (March 2019, 140 GB) database was used to capture all domains of life.

Additionally, pangenome analysis was performed for the differentially abundant MAGs between study groups using the workflow of Anvi'o v7.1 (41, 42). Gene cluster abundances were used to generate dendrograms, and pangenome analysis was performed using the --min-bit 0.5 and --mcl-inflation 10 parameters. Singletons were not considered for differential analysis and were masked in subsequent figures, as it is hard to establish the functional capacity of singletons in driving disease etiology.

### Metabolite extraction and GC-MS measurements

Stool samples were processed for homogenization based on the previous protocol, and around 50 mg of homogenized stool samples were taken for metabolite analyses (43). An internal standard mix was added to the supernatant of homogenized samples. Liquid-liquid extraction was carried out from the supernatant with cold chloroform and methanol–water. The extract was split and was vacuum dried to use further for GC-MS and LC-MS analysis. The specific analytical details for sample pre-processing, GC-MS measurements, and information about mass spectrometric data post-processing can be found in a previous publication (43). For quality control, pool samples were introduced in the measurement sequence after every fifth measurement. Compounds were annotated based on retention time and mass spectrum using an in-house mass spectral library, with an overall similarity threshold set at 0.80. Peak areas of two automatically defined quantification ions were normalized by dividing them by the peak area of the internal standard, correcting for procedural variance.

### LC-MS measurements

Metabolite analyses were conducted using a Thermo Vanquish LC coupled to a Thermo Orbitrap Exploris 240 mass spectrometer. Chromatography utilized a SeQuant ZIC-pHILIC 5 µm polymer column (150 × 2.1 mm) connected to the corresponding SeQuant ZIC-pHILIC Guard (20 × 2.1 mm) pre-column, maintained at a column temperature of 45°C. The flow rate was set at 0.2 mL/min, with mobile phases consisting of 20 mmol/L ammonium carbonate in water, pH 9.2 (eluent A), and acetonitrile (eluent B). The gradient profile was as follows: 0 min, 80% B; 3 min, 80% B; 18 min, 20% B; 19 min, 20% B; 20 min, 80% B; 24.5 min, 80%; 25.5 min, 80% B (0.4 mL/min); 29.5 min, 80% B (0.4 mL/min); 30 min, 80% B (0.2 mL/min). An injection volume of 5 µL was used. All MS experiments employed electrospray ionization with polarity switching enabled (+ESI/–ESI). Source parameters were set as follows: sheath gas flow rate, 35; aux gas flow rate, 7; sweep gas flow rate, 0; spray voltage (static), 3 kV (±); ion transfer tube temperature, 320°C; vaporizer temperature, 275°C. The Orbitrap mass analyzer operated at a resolving power of 60,000 in full-scan mode [scan range: m/z 75–1,000; radio-frequency (RF) lens, 70%, automatic gain control (AGC) target, standard; max injection time, 100 ms]. Data were acquired using Thermo Xcalibur software (Version 4.5.474.0). Data post-processing was performed with TraceFinder (Version 5.1, Build 110).

### Data analyses

All downstream analysis for the metagenome was performed using assembled contigs, and MAGs were only used for pan-genome analysis. Comparative microbiome analysis was performed for IBS and pre-GFD NCGS patient samples as well as pre-GFD (NCGS) and post-GFD (NCGS_PG) patient samples. As stated above, post-GFD samples (NCGS_PG) were analyzed solely for within-patient comparisons and were not included in the NCGS–IBS group comparisons. Statistical analyses were performed and figures generated using R v4.1.1 statistical software (https://www.R-project.org/) with phyloseq (44), ggplot2 (45), ggpubr (46), ape (47), vegan (48), and reshape2 (42) packages. Nonparametric multidimensional scaling and alpha diversity indices, such as the Shannon index, Simpson index, and observed diversity, were calculated to capture the diversity of microbial taxa, proteins, and cazymes (*P*adj. < 0.05, Wilcoxon signed-rank test). DESeq2 analysis (Wald test) with false discovery rate adjustments (Benjamini-Hochberg method) for multiple testing was used to find proteins, cazymes, KOs, and taxa significantly enriched in NCGS and IBS groups (49). The EnhancedVolcano package was used to create volcano graphs representing statistically significant differences between study groups. For metabolome analysis, the response ratios were calculated as chromatographic peak area normalized with the peak area for internal standards. The analysis refers to the relative abundance of a particular metabolite when comparing IBS and NCGS groups. PCoA was employed to assess the overall metabolite diversity between the study groups. Fold changes and log2 fold changes for the metabolites were calculated using an open-source pipeline, MetaboAnalyst v6.0 (50), and were plotted using the ggplot2 package.

## Data Availability

The data generated in the study are available at the National Center for Biotechnology Information (NCBI) BioProject Repository with ID PRJNA981497. The STORMS (Strengthening The Organizing and Reporting of Microbiome Studies) checklist is available at https://github.com/dixit-kunal/NCGS-IBS-Multiomics-Study-Data.

## SUPPLEMENTAL MATERIAL

Data S1 msphere.00856-25-s0001.xlsx Contig characteristics.

Data S2 msphere.00856-25-s0002.xlsx Metagenome bin summary, differential MAGs, and Kraken-based differential taxa.

Data S3 msphere.00856-25-s0003.xlsx List of metabolites GCMS and LCMS.

Data S4 msphere.00856-25-s0004.xlsx Differential features: metagenome analysis.

Data S5 msphere.00856-25-s0005.xlsx Disorder-specific gene clusters from pangenomes.

Figure S1 msphere.00856-25-s0006.tiff Viral diversity in NCGS and IBS.

Figure S2 msphere.00856-25-s0007.tiff Differential metagenome features between NCGS and IBS.

Figure S3 msphere.00856-25-s0008.tiff Diversity and differential abundance for metagenome features in NCGS and NCGS_PG.

Figure S4 msphere.00856-25-s0009.tiff Principal coordinate analysis for metabolite profiles.

Supplemental legends msphere.00856-25-s0010.docx Legends for Fig. S1–S4 and Data S1–S5.

ASM does not own the copyrights to Supplemental Material that may be linked to, or accessed through, an article. The authors have granted ASM a non-exclusive, world-wide license to publish the Supplemental Material files. Please contact the corresponding author directly for reuse.
