# Supplementary figures and images for: Multi-meta-omics reveal distinct microbial genomic profiles and metabolic dysregulation in non-celiac gluten sensitivity
Source: mSphere. 2026 Mar 30;11(4):e00856-25. doi: 10.1128/msphere.00856-25 (PMC13123714; doi:10.1128/msphere.00856-25)

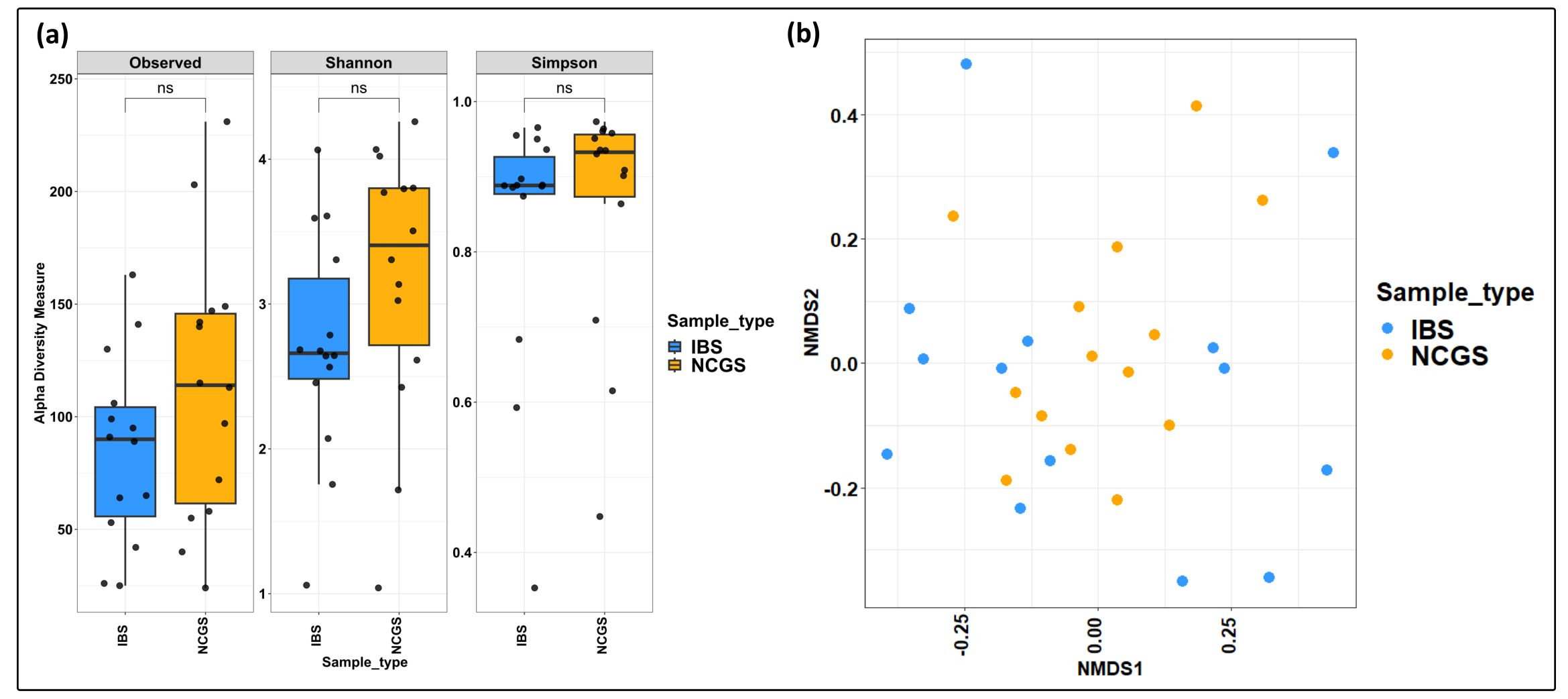

Supplement: Figure S1 — Viral diversity in NCGS and IBS. [file msphere.00856-25-s0006.tiff]

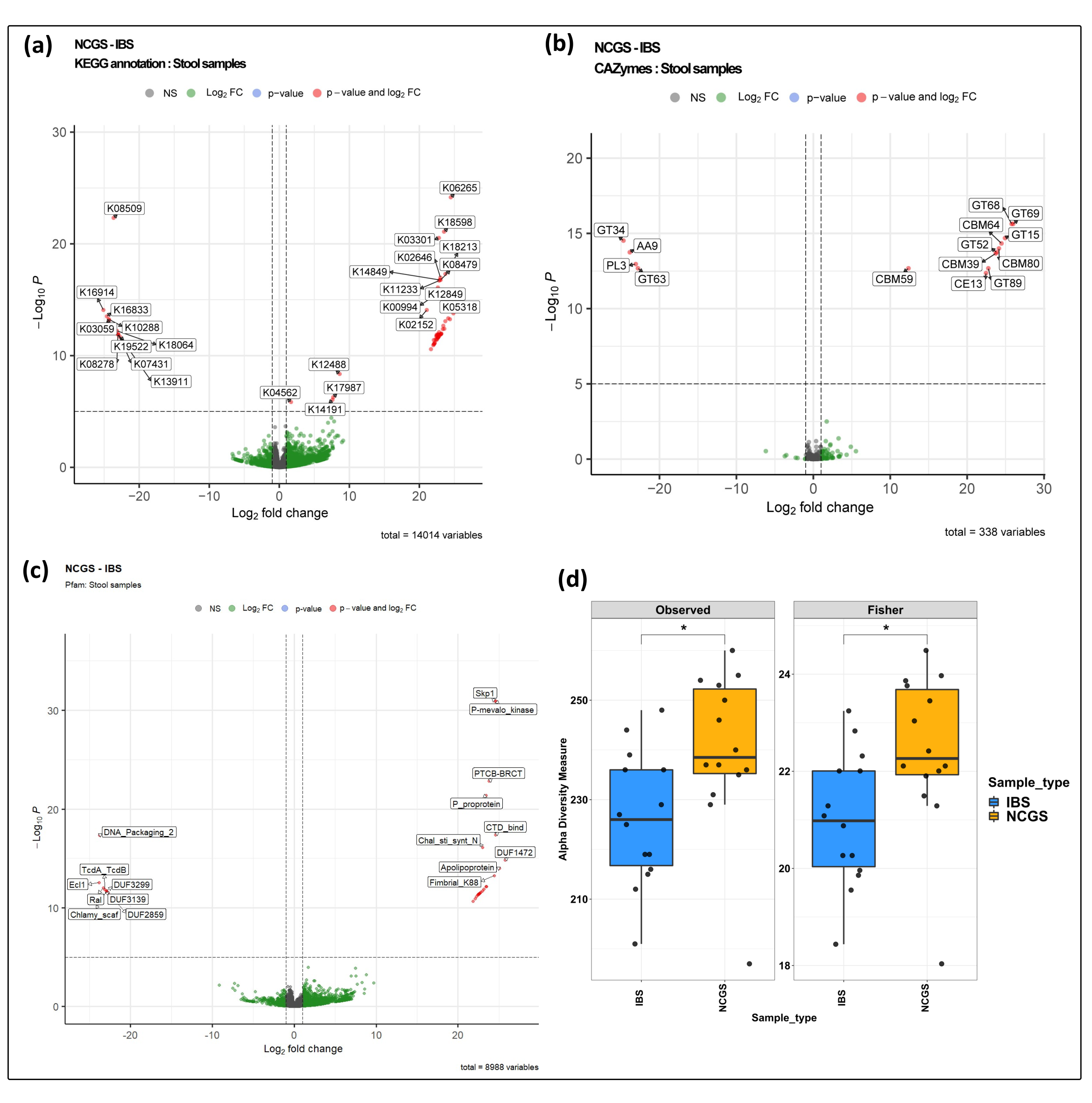

Supplement: Figure S2 — Differential metagenome features between NCGS and IBS. [file msphere.00856-25-s0007.tiff]

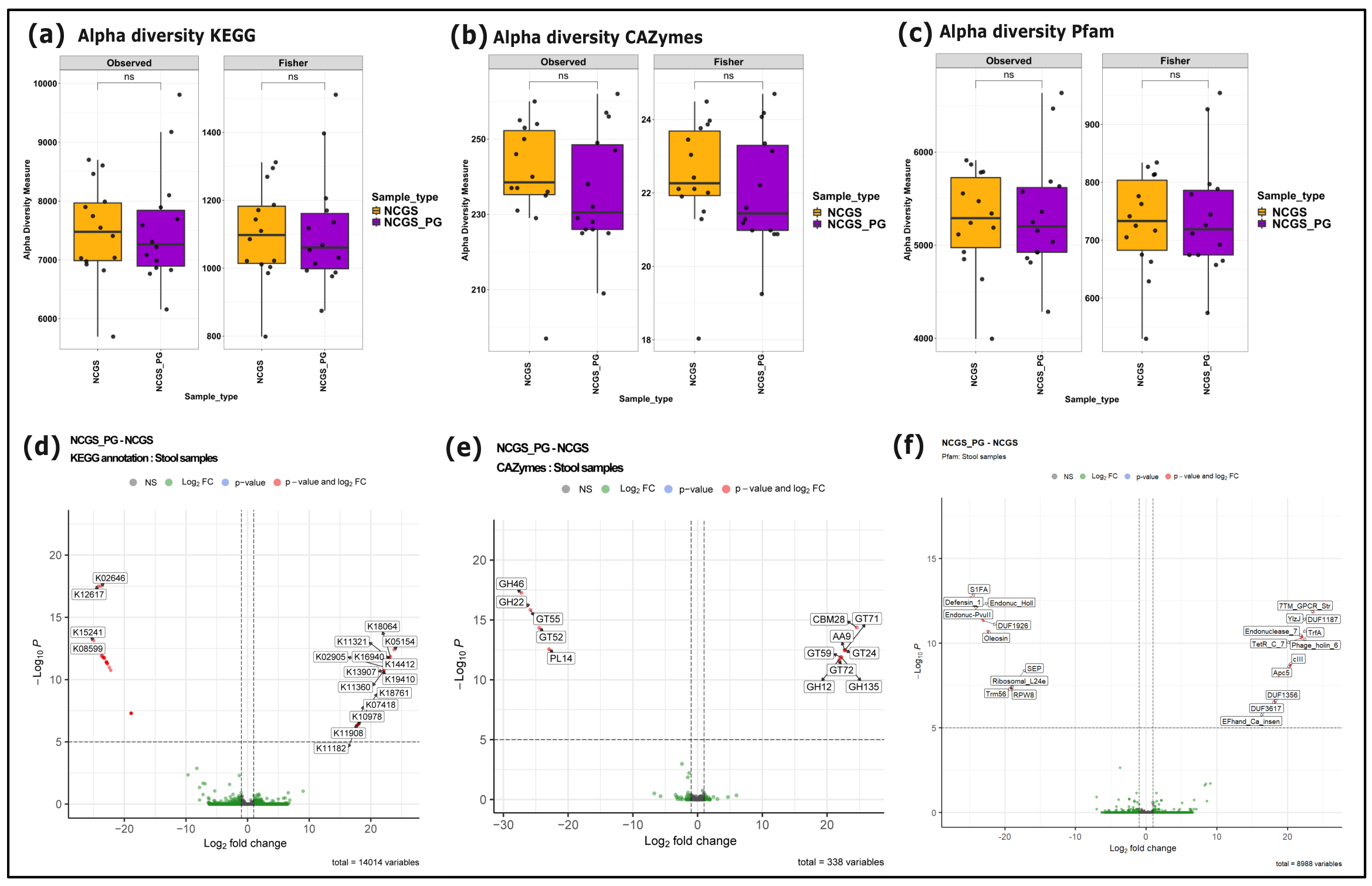

Supplement: Figure S3 — Diversity and differential abundance for metagenome features in NCGS and NCGS_PG. [file msphere.00856-25-s0008.tiff]

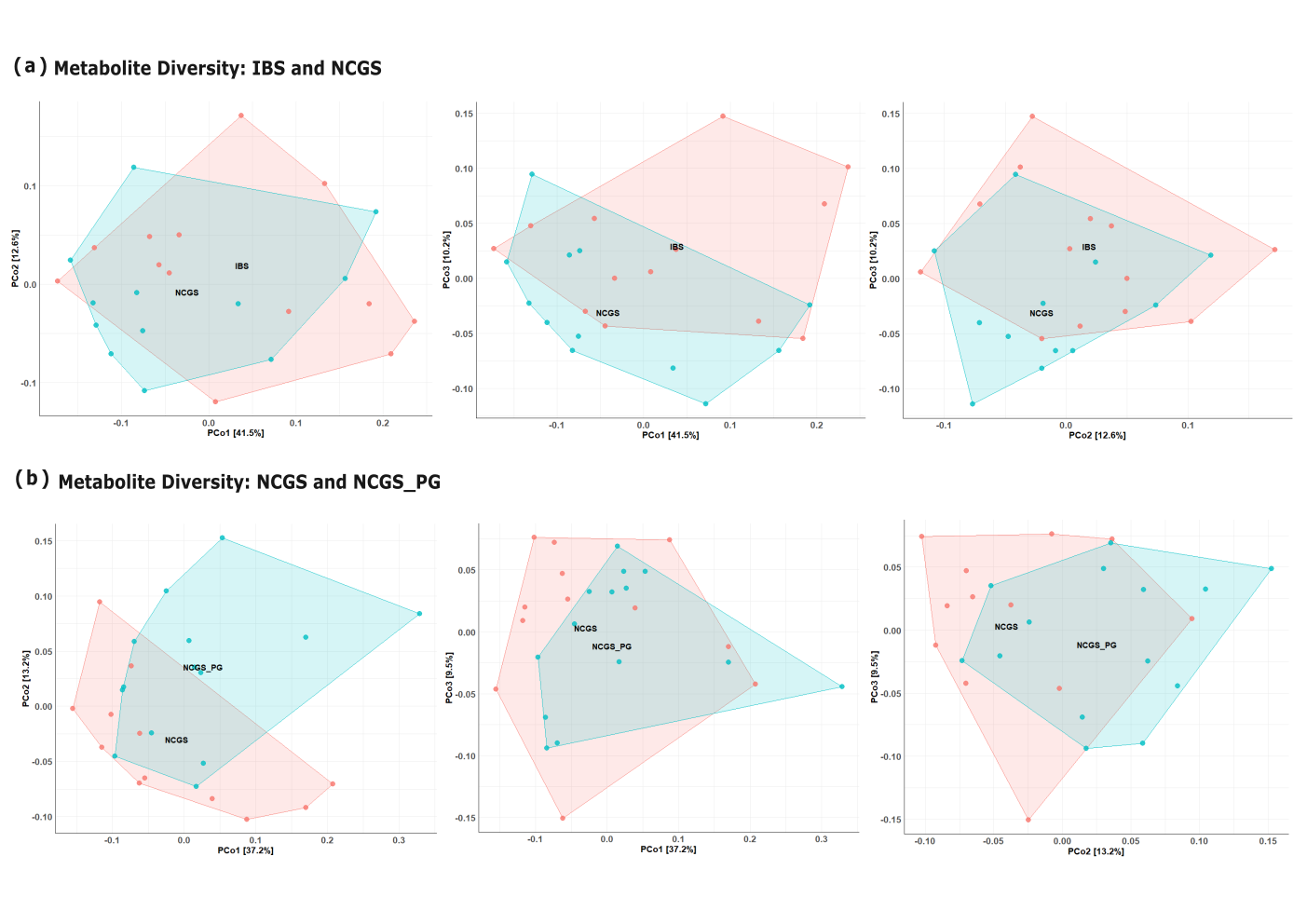

Supplement: Figure S4 — Principal coordinate analysis for metabolite profiles. [file msphere.00856-25-s0009.tiff]
