# Supplementary material for: Multi-meta-omics reveal distinct microbial genomic profiles and metabolic dysregulation in non-celiac gluten sensitivity
Source: mSphere. 2026 Mar 30;11(4):e00856-25. doi: 10.1128/msphere.00856-25 (PMC13123714; doi:10.1128/msphere.00856-25)
Supplement: Supplemental legends — Legends for Fig. S1–S4 and Data S1–S5. [file msphere.00856-25-s0010.docx]

**Multi-meta-omics reveal distinct microbial genomic profiles and metabolic dysregulation in non-celiac gluten sensitivity**

Kunal Dixit^1,2^, Susheel Bhanu Busi^3,4^, Anam Ahmed^5,6^, Avinash Kshirsagar^7^, Christian Jäger^3^, Alka Singh^5^, Varun Shah^7,8^, Sunil D. Saroj^1^, Vineet Ahuja^5^, Paul Wilmes^3^, Yogesh Shouche^7,9^, Govind Makharia^5#^, Dhiraj Dhotre^7#^

**Affiliation:**

^1^Symbiosis School of Biological Sciences, Symbiosis International (Deemed University) Pune, India

2Department of Food Science and Technology, University of California, Davis, United States of America

^3^Luxembourg Centre for Systems Biomedicine, University of Luxembourg, Esch-sur-Alzette, Luxembourg

^4^UK Centre for Ecology and Hydrology, Wallingford, Oxfordshire, United Kingdom

^5^Department of Gastroenterology and Human nutrition, All India Institute of Medical Sciences, New-Delhi, India

^6^McMaster University, Hamilton, Canada

^7^National Centre for Cell Science, Pune, India

^8^Gujarat Biotechnology University, Gandhinagar, India

^9^SKAN Research Trust, Bengaluru, India

# Corresponding Authors

**Supplementary Figure Legends:**

**Figure S1:** Viral diversity between NCGS and IBS patients overlaps. (a) Box plots depicting alpha diversity measures for viral diversity. (b) NMDS plot showing overlapping viral diversity between study groups. Comparisons shown are between IBS and pre-GFD NCGS only.

**Figure S2:** Volcano plot depicting differentially abundant (a) KOs, (b) Cazymes, and (c) protein families. (d) Box plot presenting alpha diversity measures of Cazyme abundance between NCGS and IBS patients. Comparisons shown are between IBS and pre-GFD NCGS only.

**Figure S3:** Alpha diversity measures for (a) KOs, (b) Cazymes, and (c) protein families are not significantly different between NCGS (pre GFD) and NCGS_PG (post GFD). Volcano plot depicting differentially abundant features observed between NCGS and NCGS_PG (d) KOs, (e) Cazymes, and (f) protein families. Comparisons shown are between pre-GFD NCGS and post-GFD NCGS only.

**Figure S4:** Beta diversity of metabolome profiles are overlapping for (a) NCGS and IBS patients and (b) NCGS patients before (NCGS) & after GFD (NCGS_PG). This analysis is based on all three study groups: IBS, NCGS and NCGS_PG.

**Supplementary Table Legends:**

**Supplementary data 1:** Table listing contig characteristics and alignment statistics for stool metagenomes for all three study groups: IBS, NCGS and NCGS_PG..

**Supplementary data 2:** Table listing summary for all metagenome assembled genome (MAGs) assembled from samples and significantly different MAGs between IBS - NCGS samples as well as NCGS - NCGS_PG samples. The table also lists differentially abundant bacteria, Archaea and viruses between IBS - NCGS samples as well as NCGS - NCGS_PG samples. This file presents data from all three study groups: IBS, NCGS, and NCGS_PG.

**Supplementary data 3:** Table listing metabolites identified using GCMS and LCMS along with respective response ratios (Normalized with internal standards) in IBS NCGS and NCGS_PG samples. This table lists metabolome data for all three study groups: IBS, NCGS, and NCGS_PG.

**Supplementary data 4:** Significantly different metagenomic features identified (KO, Cazymes, Protein families) between IBS and NCGS samples as well as NCGS and NCGS_PG samples. This table lists data from all three study groups: IBS, NCGS and NCGS_PG.

**Supplementary data 5:** Disorder specific gene clusters identified using pangenome analysis for *RUG115 sp900066395, Ligilactobacillus ruminis,* and *Prevotella sp003447235* genomes present in NCGS and IBS patient samples. This table lists data from the study groups NCGS and IBS.
